# Patient-specific iPSCs-derived neuronal models of spinocerebellar ataxia type 1 as a platform for studying calcium mishandling

**DOI:** 10.1016/j.gendis.2026.102215

**Published:** 2026-04-25

**Authors:** Dmitriy Grekhnev, Egor Volovikov, Olga Lebedeva, Anton Skopin, Konstantin Gusev, Sergey Klyushnikov, Alexandra Bogomazova, Guanghui Wang, Elena Zerkalenkova, Sergey Illarioshkin, Maria Lagarkova, Elena Kaznacheyeva, Vladimir Vigont

**Affiliations:** a Laboratory of Ion Channels of Cell Membranes, Institute of Cytology RAS, St. Petersburg 194064, Russia; b Laboratory of Cell Biology, Lopukhin Federal Research and Clinical Center of Physical-Chemical Medicine of Federal Medical Biological Agency, Moscow 119435, Russia; c Russian Center of Neurology and Neurosciences, Moscow 125367, Russia; d Laboratory of Molecular Neuropathology, Jiangsu Key Laboratory of Neuropsychiatric Diseases and College of Pharmaceutical Sciences, Soochow University, Suzhou, Jiangsu 215123, China; e Dmitry Rogachev National Medical Research Center of Pediatric Hematology, Oncology and Immunology, Moscow 117997, Russia; f Center for Genetic Reprogramming and Gene Therapy, Lopukhin Federal Research and Clinical Center of Physical-Chemical Medicine of Federal Medical Biological Agency, Moscow 119435, Russia; g Department of Biochemistry and Molecular Biology, Faculty of General Medicine, Pirogov Russian National Research Medical University, Moscow 117997, Russia

Spinocerebellar ataxia type 1 (SCA1) is a polyglutamine (polyQ) neurodegenerative disorder caused by the abnormal expansion of CAG repeats (> 39) in the *ATXN1* gene. This expansion leads to the production of mutant ataxin-1 protein, resulting in the formation of protein aggregates, transcriptional dysregulation, mitochondrial dysfunction and alterations in calcium handling.^1^, ^2^, ^3^ The development of effective therapies for SCA1 necessitates the identification of novel therapeutic targets. In recent years, the induced pluripotent stem cell (iPSC) technology has emerged as a transformative tool for modeling the pathogenesis of hereditary diseases and advancing personalized therapeutic strategies. Patient-derived cellular models recapitulate key pathological hallmarks of neurodegenerative diseases, including SCA1, providing a robust platform for identifying disease-specific cellular processes and conducting high-throughput drug screening.^1^, ^2^, ^3^, ^4^ However, there are only few iPSC-based neuronal models of SCA1 available to date.^1^, ^2^, ^3^ Moreover, there are no reports about SCA1-specific GABAergic medium spiny neurons (MSNs) differentiated from iPSCs. Given the shared polyQ etiology between SCA1 and Huntington's disease (HD) and our previous finding of elevated store-operated and voltage-gated calcium uptake in HD-specific MSNs,^4^ this study aimed to develop novel iPSC-based models of SCA1 and to investigate whether calcium mishandling is a convergent mechanism in polyQ diseases.

The principal scheme of SCA1-specific MSN production is shown in Figure 1A. After obtaining informed consent, dermal fibroblasts were isolated from two female patients diagnosed with SCA1. Both donors had one normal *ATXN1* allele and one allele with CAG expansion. Isolated cell lines were further denoted as SCA1.1 and SCA1.2 with 50 and 44 CAG repeats in the *ATXN1* gene, respectively (Fig. 1B; Fig. S1). Then, we reprogrammed dermal fibroblasts into a pluripotent state using lentiviral vectors to deliver Yamanaka's factors. The generated iPSCs had a normal karyotype with no chromosomal aberrations (Fig. 1C). iPSCs expressed the pluripotency markers OCT4, NANOG, SSEA-4, and TRA-1-60 (Fig. 1D). To confirm their developmental potential, we differentiated the iPSCs into cells representative of the three germ layers, as evidenced by the expression of CK18 (ectoderm), Desmin (mesoderm), and HNF4A (endoderm) (Fig. S2). Next, we used dual-SMAD inhibition to differentiate iPSCs into TUBB3- (> 90%) and DARPP32-positive (> 80%) MSNs (Fig. 1E) as previously described.^4^ A list of cell lines used in the study is provided in Table S1.

**Figure 1:**
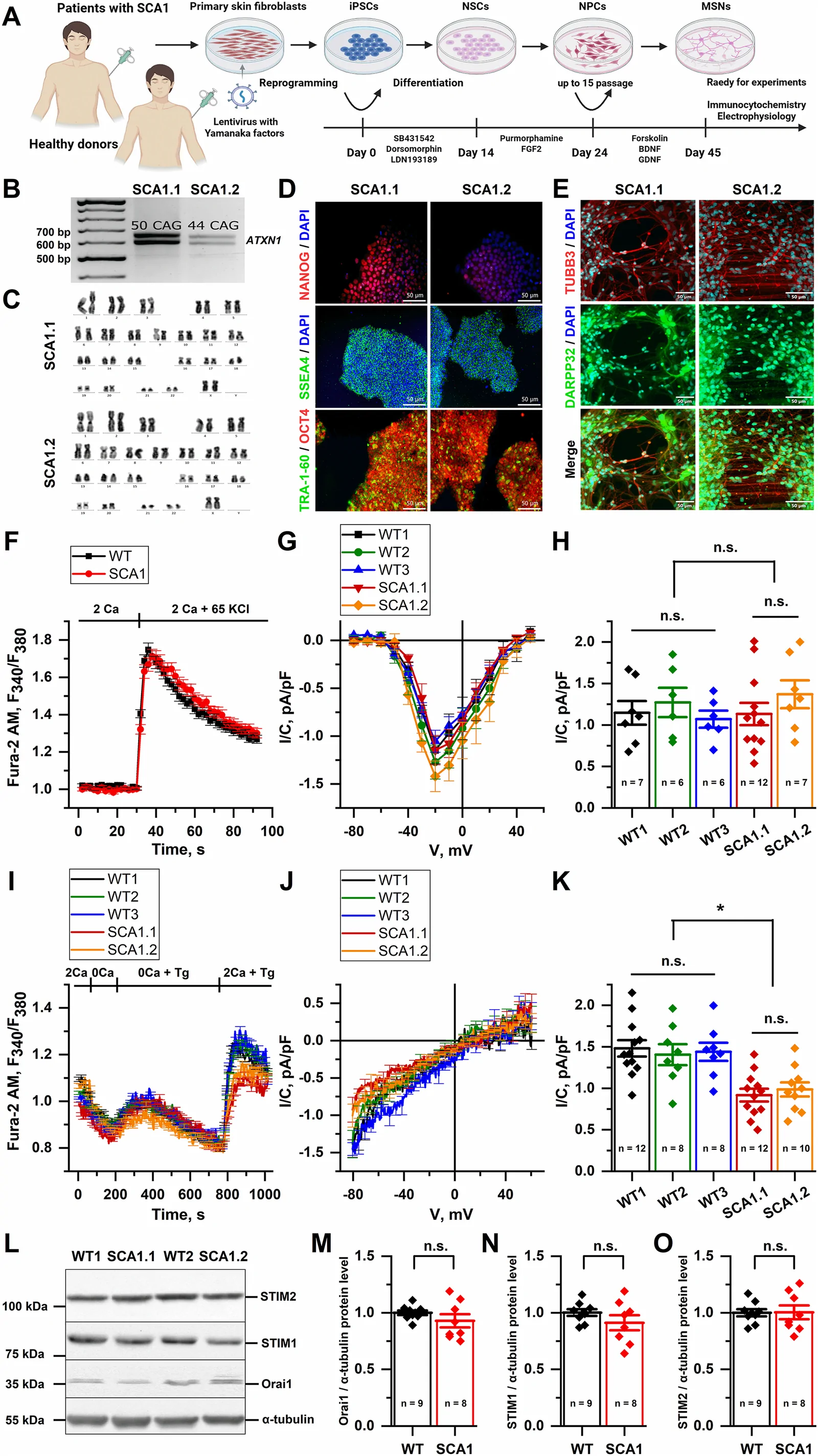
Patient-specific iPSCs-derived neuronal models of SCA1 for studying calcium mishandling. **(A)** Generation of iPSCs-based neuronal models of SCA1. The scheme was created with BioRender.com. **(B)** Agarose gel electrophoresis of PCR products amplified with primers flanking the CAG-repeat region of *ATXN1* from the DNA of SCA1.1 and SCA1.2 iPSC clones. **(C)** Karyogram of SCA1.1 and SCA1.2 iPSCs. **(D)** Immunocytochemical verification of Nanog, Oct4, SSEA-4, and TRA-1-60 expression in iPSCs of SCA1.1. and SCA1.2. Scale bar: 50 μm. **(E)** Immunocytochemical verification of TUBB3 and DARPP32 expression in MSNs generated from SCA1.1 and SCA1.2 origin. Scale bar: 50 μm. **(F)** Relative fluorescence of Fura-2 AM (F_340_/F_380_) for WT (black line) and SCA1 (red line) MSNs evoked by membrane depolarization with the application of 65 mM KCl. Curves are plotted as the mean ± SEM. Applicated solutions are indicated above the traces. Numbers of analyzed cells (*n*): WT: *n* = 298; SCA1: *n* = 287. **(G)** Average current–voltage relationships (I/V curves) of normalized voltage-gated calcium currents for WT1 (black line), WT2 (olive line), WT3 (blue line), SCA1.1 (red line) and SCA1.2 (orange line) MSNs. The number of single-cell experiments (*n*) is depicted at the panel (H). **(H)** Average amplitude of voltage-gated calcium currents at the potential of −20 mV for WT1 (black line), WT2 (olive line), WT3 (blue line), SCA1.1 (red line) and SCA1.2 (orange line) MSNs. The amplitudes are plotted as the mean ± SEM. **(I)** Relative fluorescence of Fura-2 AM (F_340_/F_380_) for WT1 (black line), WT2 (olive line), WT3 (blue line), SCA1.1 (red line) and SCA1.2 (orange line) MSNs evoked by passive depletion of calcium stores with thapsigargin (1 μM). Curves are plotted as the mean ± SEM. Applicated solutions are indicated above the traces. Numbers of analyzed cells (*n*): WT1: *n* = 112; WT2: *n* = 162; WT3: *n* = 153; SCA1.1: *n* = 148; SCA1.2: *n* = 118. **(J)** Average I/V curves of normalized currents evoked by thapsigargin (1 μM) for WT1 (black line), WT2 (olive line), WT3 (blue line), SCA1.1 (red line) and SCA1.2 (orange line) MSNs. The I/V curves were plotted after the full development of the SOC currents. The number of single-cell experiments (*n*) is depicted at the panel (K). **(K)** Average amplitudes of SOC currents at the potential of −80 mV for WT1 (black line), WT2 (olive line), WT3 (blue line), SCA1.1 (red line) and SCA1.2 (orange line) MSNs. The amplitudes are plotted as the mean ± SEM. **(L)** Representative Western blot and densitometry analysis of WT and SCA1 MSNs for Orai1 **(M)**, STIM1 **(N)** and STIM2 **(O)**. Orai1, STIM1 and STIM2 protein levels were normalized to α-tubulin (M−O). The asterisks indicate that differences in amplitudes are statistically significant (*P* < 0.05). n.s. indicates the absence of statistically significant differences (*P* > 0.05).

To validate the functional maturity of SCA1-specific MSNs, we performed a combined fluorescent and electrophysiological analysis of voltage-gated calcium channels (VGCC). We demonstrated sensitivity to membrane depolarization induced by the application of 65 mM KCl in the entire population of terminally differentiated MSNs (Fig. 1F). Measurements of the currents through VGCC revealed peak amplitudes of 1.15 ± 0.14 pA/pF, 1.27 ± 0.18 pA/pF and 1.05 ± 0.12 pA/pF in MSNs derived from iPSCs of healthy donors (WT1, WT2 and WT3) and 1.15 ± 0.15 pA/pF, 1.42 ± 0.16 pA/pF in ones derived from iPSCs of SCA1 patients (SCA1.1 and SCA1.2), respectively (Fig. 1G and H). No significant differences were observed in current amplitudes through the VGCC either among WT lines or between WT and SCA1-specific MSNs. The recorded VGCC-mediated currents were sensitive to inhibition by the L-type channel blocker nifedipine (10 μM) (Fig. S3B–S3H). Also, we confirmed the presence of tetrodotoxin-sensitive voltage-gated sodium currents (Fig. S3A).

We next investigated store-operated calcium entry (SOCE), one of the major pathways for calcium influx triggered by the depletion of endoplasmic reticulum (ER) calcium stores. SOCE is increasingly implicated in the pathogenesis of neurodegenerative diseases and represents a potential therapeutic target. To evaluate SOCE, we performed single-cell calcium imaging using the ratiometric indicator Fura-2 AM. ER calcium store depletion and subsequent SOCE activation were induced by thapsigargin (Tg; 1 μM). The results demonstrated a significant reduction in SOCE amplitude in SCA1-specific MSNs compared to controls (Fig. 1I). The SOCE mechanism was confirmed by pretreating cells for 5 min with the canonical inhibitors BTP2 (5 μM) or CM4620 (5 μM), which abolished Fura-2 fluorescence (Fig. S4A and S4B). Notably, the ER calcium content in SCA1 MSNs was not detectably altered (Fig. 1I). Electrophysiological recordings demonstrated that thapsigargin-induced SOCE amplitudes were equal to 1.48 ± 0.12 pA/pF, 1.39 ± 0.18 pA/pF and 1.45 ± 0.10 pA/pF in WT1, WT2 and WT3 MSNs and 0.93 ± 0.17 pA/pF, 1.01 ± 0.15 pA/pF in SCA1.1 and SCA1.2 MSNs, respectively (Fig. 1J and K). The recorded currents were sensitive to the widely used SOCE inhibitors 2-APB (50 μM) and Gd^3+^ (1 μM) (Fig. S4C–S4F). Thus, SOCE was significantly reduced in SCA1-specific MSNs compared with the healthy ones. To elucidate the molecular basis for the observed SOCE deficiency, we estimated the expression levels of key SOCE components: the pore-forming unit Orai1 and activators of store-operated calcium channels STIM1 and STIM2. However, no significant differences in the protein levels were detected between WT and SCA1-specific MSNs (Fig. 1L–O). At the same time, previously published studies of an SCA1 transgenic mouse model and patient brain tissue reported reduced expression of several genes encoding calcium-handling proteins, including the receptor for inositol 1,4,5-trisphosphate type 1 (*ITPR1*), the inositol polyphosphate 5-phosphatase (*INPP5A*), the SERCA2 calcium pump (*ATP2A2*) and the channel subunit TRPC3 (*TRPC3*).^5^ A comprehensive list of calcium signaling-related genes reported as downregulated in various SCA1 models is provided in Table S2. Notably, our MSN-based SCA1 model did not recapitulate the significant downregulation of certain genes (Fig. S5A) observed in Purkinje cells from mouse models and in the cerebellum of an SCA1 patient.^5^ However, we noted a trend toward TRPC3 protein downregulation in SCA1 MSNs (Fig. S5B), which may partially underlie the observed reduction in SOCE. Although the mechanistic links between CAG expansion in the *ATXN1* gene and SOCE impairment require further clarification, these early results reinforce the notion that calcium mishandling is a central pathological feature of SCA1.

While MSNs are the primary vulnerable population in HD, SCA1 is characterized predominantly by the loss of Purkinje cells in the cerebellum, with MSNs being less severely affected. However, current protocols for obtaining Purkinje cells from iPSCs suffer from complexity and poor reproducibility. Future efforts must focus on developing robust differentiation protocols for human Purkinje cells to fully capture disease-specific pathology. The abundance of calcium handling proteins in Purkinje cells reflects their importance in calcium signaling relative to other neuronal types. Therefore, even subtle changes in calcium transients might lead to Purkinje cell dysfunction and progressive degeneration in SCA1.

Nevertheless, our findings demonstrating different SOCE dysregulation in highly affected HD-specific MSNs and mildly affected SCA1-specific MSNs underscore that alterations in calcium signaling have both cell-type and disease-specific signatures across polyQ disorders, establishing elevated SOCE as a hallmark of vulnerable neurons and contributing to the “calcium hypothesis” of selective vulnerability. The unique capacity of iPSCs to be differentiated in various neuronal types opens up great perspectives for future studies focusing on the selective alterations of intracellular signaling in different types of neurons. At the same time, this implies that effective therapeutic strategies may need to be tailored to each specific polyQ disease.

In general, novel iPSC-based models of SCA1 spread the horizons of our knowledge about calcium mishandling in different neuronal types and postulate modulation of calcium channels and correction of the expression of proteins responsible for calcium homeostasis as promising directions for neurodegenerative drug development. Further investigation of these mechanisms will provide deeper insights into neurodegeneration and contribute to the advancement of patient-oriented, personalized treatment strategies.

## CRediT authorship contribution statement

**Dmitriy Grekhnev:** Writing – original draft, Visualization, Validation, Methodology, Investigation, Formal analysis, Data curation. **Egor Volovikov:** Writing – original draft, Validation, Methodology, Investigation, Formal analysis, Data curation. **Olga Lebedeva:** Writing – review & editing, Visualization, Validation, Methodology, Investigation, Formal analysis. **Anton Skopin:** Writing – review & editing, Visualization, Validation, Methodology, Investigation, Formal analysis. **Konstantin Gusev:** Writing – review & editing, Visualization, Validation, Methodology, Investigation, Formal analysis. **Sergey Klyushnikov:** Writing – review & editing, Resources, Conceptualization. **Alexandra Bogomazova:** Writing – review & editing, Supervision, Conceptualization. **Guanghui Wang:** Writing – review & editing, Supervision, Conceptualization. **Elena Zerkalenkova:** Visualization, Validation, Methodology, Investigation, Formal analysis. **Sergey Illarioshkin:** Writing – review & editing, Supervision, Conceptualization. **Maria Lagarkova:** Writing – review & editing, Supervision, Funding acquisition, Conceptualization. **Elena Kaznacheyeva:** Writing – review & editing, Supervision, Funding acquisition, Conceptualization. **Vladimir Vigont:** Writing – review & editing, Validation, Supervision, Project administration, Methodology, Investigation, Funding acquisition, Formal analysis, Conceptualization.

## Ethics declaration

This study was approved by the local ethics committees of Institute of Cytology RAS (protocol 23/23), Lopukhin Federal Research and Clinical Center of Physical-Chemical Medicine of Federal Medical Biological Agency (protocol 2021-06-01/1) and Russian Center of Neurology and Neuroscience (protocol 11/12). Written informed consents (available upon request) were obtained from all participants to donate their samples to generate the iPSCs used in the manuscript.

## Funding

The study was supported by the Grants No. 25-15-00165 (DG, AG, VV) from the Russian Science Foundation and by the Ministry of Science and Higher Education of Russian Federation (the Federal Scientific-technical programme for genetic technologies development for 2019–2030, agreement No. 075-15-2025-518) (EV, OL, AB, ML).

## Conflict of interests

The authors have no competing interests to declare.

## References

[1] Buijsen R.A.M., Hu M., Sáez-González M. (2023). Spinocerebellar *Ataxia* type 1 characteristics in patient-derived fibroblast and iPSC-derived neuronal cultures. Mov Disord.

[2] Sheeler C., Labrada E., Duvick L. (2024). Expanded ATXN1 alters transcription and calcium signaling in SCA1 human motor neurons differentiated from induced pluripotent stem cells. Neurobiol Dis.

[3] Homma H, Yoshioka Y, Fujita K (2024). Dynamic molecular network analysis of iPSC-Purkinje cells differentiation delineates roles of ISG15 in SCA1 at the earliest stage. Commun Biol.

[4] Vigont V.A., Grekhnev D.A., Lebedeva O.S. (2021). STIM2 mediates excessive store-operated calcium entry in patient-specific iPSC-derived neurons modeling a juvenile form of Huntington's disease. Front Cell Dev Biol.

[5] Lin X., Antalffy B., Kang D., Orr H.T., Zoghbi H.Y. (2000). Polyglutamine expansion down-regulates specific neuronal genes before pathologic changes in SCA1. Nat Neurosci.

