# Supplementary material for: Patient-specific iPSCs-derived neuronal models of spinocerebellar ataxia type 1 as a platform for studying calcium mishandling
Source: Genes Dis. 2026 Apr 25;14(3):102215. doi: 10.1016/j.gendis.2026.102215 (PMC13734881; doi:10.1016/j.gendis.2026.102215)
Supplement: Supplementary file 1 [file mmc1.docx]

**Supplementary data**

**Figure S1.** Sanger sequencing of the *ATXN1* locus.

Representative chromatograms and sequence alignments for SCA1.1 and SCA1.2. Chromatogram traces correspond to the analysed samples, and the aligned nucleotide sequence corresponds to the reference genome.

**Figure S2.** Differentiation of iPSCs into derivatives of the three germ layers. Immunocytochemical staining for CK18 (ectoderm), desmin (mesoderm) and HNF4A (endoderm) in embryoid body (EB) derivatives generated from SCA1.1 and SCA1.2 iPSC lines.

**Figure S3.** Pharmacological validation of voltage-gated currents in MSNs.

(**A**) Representative whole-cell recordings of voltage-gated sodium currents in MSNs, showing sensitivity to tetrodotoxin (TTX; 100 nM).

(**B**) Representative whole-cell recordings of voltage-gated calcium currents in MSNs, showing sensitivity to nifedipine (10 µM).

(**C, E, G**) Mean normalized current–voltage (I–V) relationships for voltage-gated calcium currents recorded in WT MSNs (before, black; after nifedipine, grey), SCA1.1 MSNs (before, red; after nifedipine, pink) and SCA1.2 MSNs (before, orange; after nifedipine, light orange).

(**D, F, H**) Quantification of calcium current amplitudes at −20 mV in WT, SCA1.1, and SCA1.2 MSNs before and after nifedipine (10 µM). Sample sizes (n) are indicated in the corresponding panels. The amplitudes are plotted as the mean ± SEM. The asterisks indicate that differences in amplitudes are statistically significant (p < 0.05).

**Figure S4.** Pharmacological validation of store-operated calcium currents in MSNs.

(**A, B**) Representative Fura-2 recordings of thapsigargin-induced (Tg; 1 µM) ER calcium release and SOCE in WT and SCA1 MSNs. MSNs were pretreated for 5 min with (**A**) BTP2 (5 µM) or (**B**) CM4620 (5 µM) or vehicle (DMSO; 0.1%), as indicated. Curves are plotted as the mean ± SEM. The applicated solutions are indicated above the traces.

(**C, E**) Representative thapsigargin-induced whole-cell current v.s. time courses at −80 mV in WT and SCA1 MSNs. Arrows indicate the time of (**С**) 2-APB (50 µM) or (**E**) Gd³⁺ (1 µM) application.

(**D, F**) Representative current–voltage (I–V) relationships corresponding to the time-course traces for (**С**) and (**E**) at maximal current level and after inhibitor application.

**Figure S5.** Expression of calcium signaling genes in SCA1-specific MSNs.

**(A)** Relative mRNA expression of *TRPC3*, *INPP5A*, *ATP2A2* and *ITPR1* in WT and SCA1 MSNs. Data are shown as 2^−ΔΔCt^ and were normalized to the reference genes *UBE2D2* and *RPL13A*. Bars indicate mean ± SEM, and dots denote biological replicates. Statistical significance was assessed using unpaired two-sided t-tests on ΔCt values with Benjamini–Hochberg correction; adjusted p-values are reported (ns, not significant). (**B**) Representative Western blot and quantification of TRPC3 protein level, normalized to α-tubulin.

**Table S1** iPSС lines used in the study

| **Line name** | **Type of cell line** | **Origin info** | **Method of reprogramming** | **Morphology** | **Phenotype** | **Genotype** | **Mutation analysis** | **Microbiology and virology** | **Differentiation potential** |
| --- | --- | --- | --- | --- | --- | --- | --- | --- | --- |
| **Analysis** | | |  | *Microscopy* | *RT-PCR and immunocytochemistry* | *Karyotype (G-banding)* | *PCR analysis for CAG-expansion* | *Mycoplasma testing by PCR* | *Embryoid body formation* |
| **SCA1A-27L**  **(SCA1.1)** | iPSCs | **SCA1 patient**  Age: 41  Sex: female  Ethnicity: Caucasian  **Repeats in expanded allele: 50** | Lentivirus vectors carrying the Yamanaka factors  (Oct4, Sox2, Klf4, and cMyc) | Typical morphology for human pluripotent stem cells | ICC: Oct4, SSEA4, TRA-1-60, Nanog | 46XX  Resolution: 400 bands | Heterozygote with one expanded allele | negative | ICC: CK18, Desmin, HNF4A |
| **SCA1K-2L**  **(SCA1.2)** | iPSCs | **SCA1 patient**  Age: 55  Sex: female  Ethnicity: Caucasian  **Repeats in expanded allele: 44** | Lentivirus vectors carrying the Yamanaka factors  (Oct4, Sox2, Klf4, and cMyc) | Typical morphology for human pluripotent stem cells | ICC: Oct4, SSEA4, TRA-1-60, Nanog | 46XX  Resolution: 400 bands | Heterozygote with one expanded allele | negative | ICC: CK18, Desmin, HNF4A |
| **IPSRG4S**  **(WT1)** | iPSCs | **Healthy donor**  Age: 60 years  Sex: male  Ethnicity: Caucasian | Sendai virus vectors carrying the Yamanaka factors  (Oct4, Sox2, Klf4, and cMyc) | Vigont et al., 2021 | | | | | |
| **IPSRG2L**  **(WT1)** | iPSCs |  | Lentivirus vectors carrying the Yamanaka factors  (Oct4, Sox2, Klf4, and cMyc) | Vigont et al., 2021 | | | | | |
| **FF-2S**  **(WT2)** | iPSCs | **Healthy donor**  Age: 48 years  Sex: female  Ethnicity: Caucasian | Sendai virus vectors carrying the Yamanaka factors  (Oct4, Sox2, Klf4, and cMyc) | Bogomiakova et al., 2021 | | | | | |
| **FD-3.9L**  **(WT3)** | iPSCs | **Healthy donor**  Age: 26 years  Sex: female  Ethnicity: Caucasian | Lentivirus vectors carrying the Yamanaka factors  (Oct4, Sox2, Klf4, and cMyc) | Vigont et al., 2021 | | | | | |

**Table S2** List of downregulated calcium signaling related genes in SCA1 models.

| Genes | Proteins | Models | References |
| --- | --- | --- | --- |
| *ITPR1* | IP_3_-receptor type 1 | Purkinje neurons of transgenic mice  Cerebellum and pons of a SCA1 patient | Lin et al., 2000  Serra et al. 2004  Chopra et al., 2020  Takahashi et al. 2022 |
| *ITPR2* | IP_3_-receptor type 2 | iPSC-derived human motoneurons | Sheeler et al., 2024 |
| *INPP5A* | Inositol polyphosphate 5-phosphatase type 1 | Purkinje neurons of transgenic mice | Lin et al., 2000 |
| *ATP2A2* | SERCA2 | Purkinje neurons of transgenic mice  Cerebellum and pons of a SCA1 patient | Lin et al., 2000 |
| *ATP2A3* | SERCA3 | Purkinje neurons of transgenic mice | Serra et al. 2004 |
| *TRPC3* | TRPC3 | Purkinje neurons of transgenic mice | Lin et al., 2000  Chopra et al., 2020 |
| *SYT15* | Synaptotagmin | iPSC-derived human motoneurons | Sheeler et al., 2024 |
| *CALB1* | Calbindin | Purkinje neurons of transgenic mice  Cerebellum of SCA1 patients | Vig et al. 1996  Vig et al. 1998  Serra et al. 2004  Takahashi et al. 2022 |
| *PVALB* | Parvalbumin | Purkinje neurons of transgenic mice  Cerebellum of SCA1 patients | Vig et al. 1996  Vig et al. 1998 |
| *CACNA1G* | Cav3.1, T-type | Purkinje neurons of transgenic mice | Serra et al. 2004  Chopra et al., 2020 |
| *GRM1* | mGluR1 | Purkinje neurons of transgenic mice | Serra et al. 2004  Chopra et al., 2020 |
| *HOMER3* | Homer-3 | Purkinje neurons of transgenic mice | Serra et al. 2004 |
| *SLC1A6* | EAAT4, Excitatory amino acid transporter 4 | Purkinje neurons of transgenic mice | Lin et al., 2000  Serra et al. 2004 |
| *KCNMA1* | Calcium-activated BK channel | Purkinje neurons of transgenic mice | Chopra et al., 2020 |

**Table S3** Antibodies

| ANTIGEN,  FUNCTION | HOST SPECIES | DILUTION | COMPANY CAT # and RRID |
| --- | --- | --- | --- |
| NANOG,  Pluripotency marker | Rabbit | 1:100 | ThermoFischer Cat# MA1-017 |
| SSEA 4,  Pluripotency marker | Mouse | 1:50 | DSHB Cat# MC81370 |
| TRA-1-60,  Pluripotency marker | Mouse | 1:500 | Abcam Cat# ab16288 |
| OCT4,  Pluripotency marker | Rabbit | 1:100 | Abcam Cat# ab18976 |
| Desmin,  Mesoderm marker | Rabbit | 1:500 | Abcam Cat# ab15200 |
| HNF4α,  Endoderm marker | Rabbit | 1:200 | Abcam Cat# ab92378 |
| CK18, Ectoderm marker | Mouse | 1:100 | DAKO Cat# M7010 |
| TUBB3,  Neuronal marker | Mouse | 1:200 | ABclonal Cat# A18132 |
| DARPP-32,  Marker of GABA-ergic neurons | Rabbit | 1:100 | Abcam Cat# ab40801 |
| Secondary Alexa488-anti Rabbit | Goat | 1:1000 | Abcam Cat# 181448 |
| Secondary Alexa555- anti Mouse | Goat | 1:1000 | Thermo Fisher Cat# A21424 |
| STIM1 | Rabbit | 1:1000 | Cell Signaling Cat# 5668S |
| STIM2 | Rabbit | 1:1000 | Cell Signaling Cat# 4917S |
| Orai1 | Mouse | 1:1000 | Sigma Cat# SAB4200273 |
| TRPC3 | Rabbit | 1:1000 | Cell Signaling Cat# 77934S |
| α-tubulin | Mouse | 1:1000 | Sigma Cat# T6074 |
| Anti-Mouse IgG (Fc specific)–Peroxidase antibody produced in goat | Goat | 1:30000 | Sigma Cat# A0168 |
| Anti-Rabbit IgG (whole molecule)–Peroxidase antibody produced in goat | Goat | 1:30000 | Sigma Cat# A0545 |

**Table S4** Primer sequences

| TARGET | APPLICATION | FORWARD / REVERSE PRIMERS 5’-3’ |
| --- | --- | --- |
| *ATXN1* | CAG-expansion validation | F: CACCACTCCATCCCAGCG  R: ACGTACTGGTTCTGCTGGGC |
| *UBE2D2* | Reference gene | F: CTACGATCACAGTGGTCTCCAG  R: CGAGCAATCTCAGGCACTAAAGG |
| *RPL13A* | Reference gene | F: CTCAAGGTGTTTGACGGCATCC  R: TACTTCCAGCCAACCTCGTGAG |
| *TRPC3* | Gene of interest | F: GGGGCTAAAGTTAATGCTGCT  R: CATTCTACATCACTGTCATCCTCAA |
| *INPP5A* | Gene of interest | F: GCTCTTCGACGACCCAGAAA  R: AGGTAGACTCGAGCCCTGTT |
| *ATP2A2* | Gene of interest | F: TAGAACGTGCAAATGCCTGC  R: CTTCAGGAGCACCCTTCACA |
| *ITPR1* | Gene of interest | F: AACACCACCATCCAGCACTC  R: TGATCCCGGACCTCTTCTGT |

**Methods**

**Fibroblasts isolation and cultivation**

Written informed consents were obtained from all participants (forms available upon request) for the donation of skin samples for iPSCs generation. A skin punch biopsy was collected from the forearm and dissected into small fragments under sterile conditions. Tissue pieces were placed in Petri dishes, secured under a coverslip and maintained for 14 days in DMEM (PanEco, Russia) supplemented with 10% fetal bovine serum (FBS; Gibco, USA), 1× non-essential amino acids (NEAA; PanEco, Russia) and 1× penicillin–streptomycin (PanEco, Russia). Outgrowing fibroblasts were dissociated with 0.25% trypsin (Gibco, USA) and expanded in the same medium.

**iPSCs generation and cultivation**

Patient-specific iPSCs were generated by lentiviral reprogramming of human dermal fibroblasts using OCT4, SOX2, KLF4 and c-MYC, as previously described (Nekrasov et al., 2016; Vigont et al., 2021). iPSCs were maintained on hESC-qualified Matrigel (Corning, USA) in mTeSR1 medium (STEMCELL Technologies, Canada), with daily medium changes. Cells were passaged using 0.05% trypsin–EDTA (Gibco, USA). For 24 h after passaging, the culture medium was supplemented with 5 µM Y-27632. The list of cell lines used in our study is presented in Table S1.

**Embryoid body formation**

Embryoid bodies (EBs) were generated from iPSCs using the AggreWell 400 system (STEMCELL Technologies, Canada) according to the manufacturer’s instructions. Following aggregation, EBs were transferred to ultra-low-attachment plates and cultured in DMEM/F12 supplemented with 15% KnockOut Serum Replacement (SR; Gibco, USA), 5% FBS, 0.1 mM β-mercaptoethanol (Sigma, USA), 1% NEAA and 1× penicillin–streptomycin. Medium was replaced every 2 days. After 10 days in suspension, EBs were plated onto gelatin-coated Petri dishes and maintained for an additional 15–20 days in the same medium.

**Karyotyping**

Colcemid was added to iPSCs cultures to a final concentration of 0.1 μg/ml for 20 min. Cells were then washed twice with PBS, dissociated with 0.05% trypsin and transferred to a 15-ml tube. Hypotonic treatment was performed by adding five volumes of 0.075 M KCl, followed by incubation for 20 min at 42 °C. Next, 1/40 volumes of 6:1 methanol/glacial acetic acid mix was added to cell suspension and centrifuged for 5 minutes at 400g. Cell pellet was resuspended in 6:1 methanol/glacial acetic acid mix and centrifuged for 5 minutes at 400g. Cell pellet was resuspended in 3:1 methanol/glacial acetic acid mix, centrifuged for 5 minutes at 400g. Cell pellet was resuspended in 100 µL 3:1 methanol/glacial acetic acid mix. Karyotype analysis was performed at the Dmitry Rogachev National Medical Research Center of Pediatric Hematology, Oncology and Immunology (Moscow, Russia).

**Neuronal differentiation**

Neurons were obtained as previously described (Vigont et al., 2021). Briefly, iPSCs were grown to full confluence and differentiated for 14 days in DMEM/F12 (Gibco, USA) supplemented with 1× penicillin–streptomycin, 1× GlutaMAX (Gibco, USA), 1× N2 supplement (Gibco, USA), 2% SR, 2 µM dorsomorphin (Miltenyi Biotec, Germany), 10 µM SB431542 (Miltenyi Biotec, Germany) and 1 µM LDN-193189 (Miltenyi Biotec, Germany). After that, cells were passaged by Versene solution (PanEco, Russia) to Matrigel-coated plates in 1:2 ratio, and cultures were supplemented with 5 µM Y-27632 for 24 h after passaging. Then cells were cultured for 10  days in a 1:1 mixture of DMEM/F12 and Neurobasal medium (Gibco, USA) supplemented with 1× penicillin–streptomycin, 1× GlutaMAX, 1× N2, 1× B27 (Gibco, USA), 2% SR, 20 ng /ml FGF2 (Miltenyi Biotec, Germany) and 2 µM purmorphamine (Miltenyi Biotec, Germany), with daily medium changes. At this stage, neuronal progenitor cells (NPCs) were cryopreserved to establish an NPC bank. NPCs were expanded and stored up to 15 passages. For neuronal maturation, NPCs were seeded at 4 × 10⁵ cells /cm² on Matrigel-coated plates and cultured for 21 days in Neurobasal medium supplemented with 1× penicillin–streptomycin, 1× GlutaMAX, 1× B27, 20 ng /ml BDNF (Miltenyi Biotec, Germany), 20 ng/ ml GDNF (Miltenyi Biotec, Germany) and 5 µM forskolin (Miltenyi Biotec, Germany). Medium was replaced every 3 days.

**Immunocytochemistry**

For immunocytochemical staining, cells were washed with warm PBS and fixed in 4% PFA for 15 min. They were then washed with PBS three times for 5 min each, permeabilized with 0.1% Triton X-100 solution for 15 min, washed again with PBS, and incubated for 1 hour at RT in a blocking solution consisting of PBS, 5% FBS, 5% goat Serum and 0,1% Tween-20. Primary antibodies were added in blocking solution according to dilution table (Table S3) and incubated overnight at 4°C. Next, cells were washed three times with PBST (PBS with 0.1% Tween-20), and secondary antibodies diluted in PBST were added for 1 hour at RT. Next, cells were washed three times for 5 min each with PBST, stained with a 200 ng/ml DAPI solution, and washed again with PBST. Cells were visualized under an inverted fluorescent microscope (Olympus IX53, Japan). Immunostained neurons were visualized using a confocal microscope (Olympus FV3000, Japan) as z-stacks. Images were processed with cellSens Dimensions software (Olympus). All confocal images presented are maximum-intensity projections of the z-stacks. To quantify neuronal identity, the proportion of DARPP-32^+^ / TUBB3^+^ cells was determined across ≥ 5 random fields per sample using the Fiji Cell Counter plugin.

**RNA isolation and RT–qPCR**

Total RNA was isolated using TRIzol reagent (Thermo Fisher Scientific, USA) according to the manufacturer’s instructions. cDNA was synthesized using an MMLV reverse transcriptase kit (Evrogen, Russia) following the manufacturer’s protocol. Quantitative RT–PCR was performed using qPCR-HS-SYBR mix (Evrogen, Russia) according to the manufacturer’s instructions. Relative gene expression was calculated using the ΔΔCt method. Ct values were normalized to the geometric mean of the reference genes *UBE2D2* and *RPL13A* (ΔCt). ΔΔCt values were obtained by normalizing to the mean ΔCt of the WT group. Data are presented as fold change (2^⁻ΔΔCt^). Statistical significance was assessed using unpaired t-tests on ΔCt values with Benjamini–Hochberg correction for multiple comparisons.

**DNA extraction and PCR**

Genomic DNA was extracted from cell pellets using a magnetic bead–based kit (Syntol, Russia) according to the manufacturer’s instructions. To verify the *ATXN1* CAG repeat expansion, the repeat-containing locus was amplified using previously described *ATXN1* primers flanking the CAG tract (Table S4). PCR was performed with an initial denaturation at 95 °C for 5 min, followed by 30 cycles of 95 °C for 30 s, 60 °C for 20 s and 72 °C for 30 s, and a final extension at 72 °C for 2 min.

**Sanger sequencing**

Genomic DNA from SCA1.1 and SCA1.2 was amplified using previously described (Table S4) *ATXN1* primers flanking the CAG repeat region. PCR products were resolved by agarose gel electrophoresis to separate alleles. Bands corresponding to the expanded (higher-molecular-weight) alleles were excised and purified using the Cleanup Mini kit (Evrogen, Russia) according to the manufacturer’s instructions. Purified amplicons were cloned into the pAL2-T vector (Evrogen, Russia) and transformed into *E. coli*. Recombinant colonies were identified by blue–white screening, expanded in liquid culture and plasmid DNA was isolated using a Plasmid Miniprep kit (Evrogen, Russia). Sanger sequencing was performed with the universal M13 forward primer by the Evrogen sequencing facility (Russia). Chromatograms were aligned to the human reference genome.

**Electrophoresis and Western Blotting**

Cells were cultured in 30-mm dishes and lysed in RIPA buffer containing 20 mM Tris-HCl (pH 7.6), 150 mM NaCl, 1 mM EDTA-Na, 1 mM EGTA-Na, 1% Triton X-100, 0.2 mM phenylmethylsulfonyl fluoride (PMSF) and 1× protease inhibitor cocktail (Roche). Proteins were separated by SDS–PAGE (Laemmli) on 8% polyacrylamide gels and transferred to nitrocellulose membranes (Bio-Rad, USA) using a Trans-Blot system (WEX, China). Transfer was performed in buffer containing 48 mM Tris, 39 mM glycine and 20% methanol. Membranes were blocked in 5% non-fat dry milk for 1 h at room temperature and incubated with primary antibodies followed by HRP-conjugated secondary antibodies. Proteins were visualized using SuperSignal West Femto Maximum Sensitivity Substrate (Thermo Fisher Scientific, USA). α-tubulin served as a loading control. Band intensities were quantified by densitometry using standard image analysis software (Bio-Rad, USA). A complete list of antibodies is provided in Table S3.

**Fluorescent Imaging**

Fluorescent calcium imaging was performed according to previously established protocols (Nekrasov et al., 2016; Oskolova et al., 2024). MSNs grown on 4 × 4 mm coverslips were loaded with 2 µM Fura‑2 AM (Invitrogen, USA) in the presence of 0.025% Pluronic F‑127 and incubated in the dark at room temperature for 1 h. Cells were then washed with HBSS for 20 min. Imaging was conducted using alternating 340 nm and 380 nm excitation light at 0.5 Hz, and emission fluorescence intensity was measured at 510 nm. Changes in cytosolic calcium concentration were expressed as the ratio of emission intensities at 340 nm and 380 nm excitation (F340/F380).

VGCC‑mediated responses were evoked by applying HBSS supplemented with 65 mM KCl to depolarize the plasma membrane.

SOCE was assessed using 1 µM thapsigargin and a standard protocol in which cells were initially perfused with calcium‑free HBSS, followed by reintroduction of 2 mM CaCl₂. This approach allowed clear discrimination between calcium release from intracellular stores and subsequent calcium influx through store‑operated calcium channels.

To prevent SOCE the pretreatment of cell with canonical inhibitors BTP2 (5 µM) or CM4620 (5 µM) was used.

**Electrophysiological Recordings**

Ion currents were recorded using the whole-cell patch clamp technique as previously described (Nekrasov et al. 2016). The pipette solution contained (in mM) 125 CsCl, 10 EGTA-Cs, 10 HEPES-Cs, 4.5 CaCl_2_, 1.5 MgCl_2_, 4 Mg-ATP and 0.4 Na-GTP; pH was adjusted to 7.3 with CsOH. The extracellular solution contained (in mM) 140 NMDG-Asp, 10 BaCl_2_, 10 HEPES-Cs and 10 glucose; pH was adjusted to 7.4 with CsOH. To record currents through VGCC, we maintained cells at −40 mV and applied a series of 200 ms voltage steps, from −80 to +50 mV with an increment of 10 mV. To record currents through SOC channels, the membrane potential was initially held at −40 mV. It was then periodically (every 5 s) decreased to −100 mV for 30 ms, then gradually raised to 100 mV at a rate of 1 mV/ms and then returned to −40 mV. The recorded currents were normalized relative to cell capacitance (5–15 pF). The traces recorded prior to current activation were used as templates for leak subtraction. Currents were evoked by the application of 1 μM thapsigargin to the external solution. The same solutions as for the voltage-gated current recordings were used, with the addition of 10 μM nifedipine into the extracellular solution to block the L-type VGCC. All chemicals were obtained from Sigma-Aldrich (USA). To inhibit SOCE in the acute experiments the application of canonical store-operated channels blockers 2-APB (50 µM) or Gd^3+^ (1 µM) was used.

Sodium currents were recorded using the whole-cell patch-clamp technique. The pipette solution contained (in mM): 120 CsCl, 10 HEPES-Cs, 10 EGTA-Cs, 1.5 MgCl_2_, 4 Mg-ATP, 0.4 Na-GTP, 5.434 mM CaCl_2_, pH was adjusted to 7.3 with CsOH. External solution contained (in mM): 145 NaCl, 2 CaCl_2_, 1 MgCl_2_, 10 HEPES-Na, pH was adjusted to 7.4 with NaOH. Sodium currents were assessed by applying a 30 ms square depolarization pulse from a holding potential of −100 mV to 40 mV with 10 mV increment. Tetrodotoxin sensitivity was confirmed by repeating recordings in the presence of TTX (100 nM). The recorded sodium currents were normalized to cell capacitance.

Recordings were made in the WinWCP v5.6.7 program (The University of Strathclyde, UK). The data were processed in the Clampfit 10.6 (Molecular devices, USA).

**Statistics**

Statistical analysis was performed using Origin 8.0 (Origin Lab). The amplitudes of currents were compared using one-way ANOVA (p < 0.05) with post hoc Bonferroni correction. The normality of the distribution and the equality of variances were evaluated using the Shapiro-Wilk and Leven tests, respectively. We used the Mann-Whitney-Wilcoxon test to compare the expression levels of the proteins.

**References**

Bogomiakova ME, Sekretova EK, Eremeev AV, et al. Derivation of induced pluripotent stem cells line (RCPCMi007-A-1) with inactivation of the beta-2-microglobulin gene by CRISPR/Cas9 genome editing. *Stem Cell Res*. 2021; 55:102451.

Chopra R, Bushart DD, Cooper JP, et al. Altered Capicua expression drives regional Purkinje neuron vulnerability through ion channel gene dysregulation in spinocerebellar ataxia type 1. *Hum Mol Genet*. 2020; 29(19):3249-3265.

Lin X, Antalffy B, Kang D, Orr HT, Zoghbi HY. Polyglutamine expansion down-regulates specific neuronal genes before pathologic changes in SCA1. *Nat Neurosci.* 2000; 3(2):157-63.

Nekrasov ED, Vigont VA, Klyushnikov SA, et al. Manifestation of Huntington's disease pathology in human induced pluripotent stem cell-derived neurons. *Mol Neurodegener.* 2016; 11:27.

Oshkolova AA, Grekhnev DA, Kruchinina AA, et al. Comparison of the calcium signaling alterations in GABA-ergic medium spiny neurons produced from iPSCs of different origins. *Biochimie.* 2024; 222:63-71.

Serra HG, Byam CE, Lande JD, et al. Gene profiling links SCA1 pathophysiology to glutamate signaling in Purkinje cells of transgenic mice. *Hum Mol Genet*. 2004; 13(20):2535-43.

Sheeler C, Labrada E, Duvick L, et al. Expanded ATXN1 alters transcription and calcium signaling in SCA1 human motor neurons differentiated from induced pluripotent stem cells. *Neurobiol Dis*. 2024; 201:106673.

Takahashi T, Stoiljkovic M, Song E, et al. LINE-1 activation in the cerebellum drives ataxia. *Neuron*. 2022; 110(20):3278-3287.e8.

Vig PJ, Fratkin JD, Desaiah D, Currier RD, Subramony SH. Decreased parvalbumin immunoreactivity in surviving Purkinje cells of patients with spinocerebellar ataxia-1. *Neurology*. 1996; 47(1):249-53.

Vig PJ, Subramony SH, Burright EN, et al. Reduced immunoreactivity to calcium-binding proteins in Purkinje cells precedes onset of ataxia in spinocerebellar ataxia-1 transgenic mice. *Neurology*. 1998; 50(1):106-13.

Vigont VA, Grekhnev DA, Lebedeva OS, et al. STIM2 Mediates Excessive Store-Operated Calcium Entry in Patient-Specific iPSC-Derived Neurons Modeling a Juvenile Form of Huntington's Disease. *Front Cell Dev Biol.* 2021; 9:625231.
